# Spatial Distribution, Sources Apportionment and Health Risk of Metals in Topsoil in Beijing, China

**DOI:** 10.3390/ijerph13070727

**Published:** 2016-07-20

**Authors:** Chunyuan Sun, Wenji Zhao, Qianzhong Zhang, Xue Yu, Xiaoxia Zheng, Jiayin Zhao, Ming Lv

**Affiliations:** 1 Urban Environmental Process and Digital Modeling Laboratory, Capital Normal University, Beijing 100048, China; cysun0419@126.com (C.S.); huoguozhujiu@163.com (Q.Z.); yx150972@163.com (X.Y.); zhengxx115@163.com (X.Z.); 2 College of Environmental Sciences and Engineering, Peking University, Beijing 100871, China; jiayin.zhao@pku.edu.cn; 3 Medical Engineering Department, The General Hospital of Chinese People’s Armed Police Forces, Beijing 100039, China; yxgcbj@163.com

**Keywords:** urban topsoil, metals, spatial analysis, source analysis, health risk

## Abstract

In order to acquire the pollution feature and regularities of distribution of metals in the topsoil within the sixth ring road in Beijing, a total of 46 soil samples were collected, and the concentrations of twelve elements (Nickel, Ni, Lithium, Li, Vanadium, V, Cobalt, Co, Barium, Ba, Strontium, Sr, Chrome, Cr, Molybdenum, Mo, Copper, Cu, Cadmium, Cd, Zinc, Zn, Lead, Pb) were analyzed. Geostatistics and multivariate statistics were conducted to identify spatial distribution characteristics and sources. In addition, the health risk of the analyzed heavy metals to humans (adult) was evaluated by an U.S. Environmental Protection Agency health risk assessment model. The results indicate that these metals have notable variation in spatial scale. The concentration of Cr was high in the west and low in the east, while that of Mo was high in the north and low in the south. High concentrations of Cu, Cd, Zn, and Pb were found in the central part of the city. The average enrichment degree of Cd is 5.94, reaching the standard of significant enrichment. The accumulation of Cr, Mo, Cu, Cd, Zn, and Pb is influenced by anthropogenic activity, including vehicle exhaustion, coal burning, and industrial processes. Health risk assessment shows that both non-carcinogenic and carcinogenic risks of selected heavy metals are within the safety standard and the rank of the carcinogenic risk of the four heavy metals is Cr > Co > Ni > Cd.

## 1. Introduction

Urban topsoil is a significant component of the urban ecological system. With rapid urbanization, plenty of toxic and hazardous metals have contaminated soils, causing serious soil pollution and decrease of capacity [1,2,3]. In 2014, the Ministry of Environmental Protection of the People’s Republic of China (MEPC) and Ministry of Land and Resources of the People’s Republic of China (MLRPC) issued an investigation result indicating that heavy metals are the most serious pollutants. Due to different land use types and human interference, the concentrations and sources of metals in urban topsoil differ and vary markedly over the space [4,5,6,7,8,9,10]. Metals in topsoil originate from natural and anthropogenic sources. Anthropogenic sources are mainly attributed to industrial activity, agricultural production, vehicle exhaust, coal combustion, waste disposal, atmospheric deposition, and other human activities [11,12,13,14,15,16,17]. Such accumulation has brought great harm to the urban environment and human health [18,19,20]. Metals such as Cd, Co, Cr, and Ni have carcinogenic effects, leading to functional disorders and irreversible damage to the human body [21,22,23]. Previous studies show that substantial accumulation of Cd in the human body can inhibit the activity of many enzymes and stimulate the human gastrointestinal system, affect bone metabolism, and induce cancer, etc. Cr (VI) is a kind of strong oxidizer that is easily absorbed by the body. Its excessive accumulation can cause respiratory system and skin diseases, etc. Nowadays, metal pollution in urban soil has become a serious problem world-wide and has attracted the attention of a large number of scientists. Hence, this study intends to visualize the spatial distribution of urban topsoil metals by geo-statistics. Based on that, the origin of the metals was identified through principal component analysis. Then, a U.S. Environmental Protection Agency model was used for health risk assessment from different exposure pathways. This research is supposed to provide references for environmental risk warning, management, and restoration. 

## 2. Data and Method

### 2.1. Study Area and Sampling

Beijing, located on the northwest edge of the North China Plain, is surrounded by Hebei Province and Tianjin. The overall terrain is high in the northwest and low in the southeast. The area is influenced by the typical warm temperate semi-humid continental monsoon climate, with four distinct seasons. North or northwest winds dominate in the winter, and south or southeast winds prevail in the summer. The total area is 16,410.54 km^2^ with 16 districts. The study area covers all places inside the sixth ring road. The main soil type of our study area is cinnamon soil and moisture soil. The land use types include urban land, urban built-upland, forest land, grassland, garden land, and other building land, etc.

**Figure 1:**
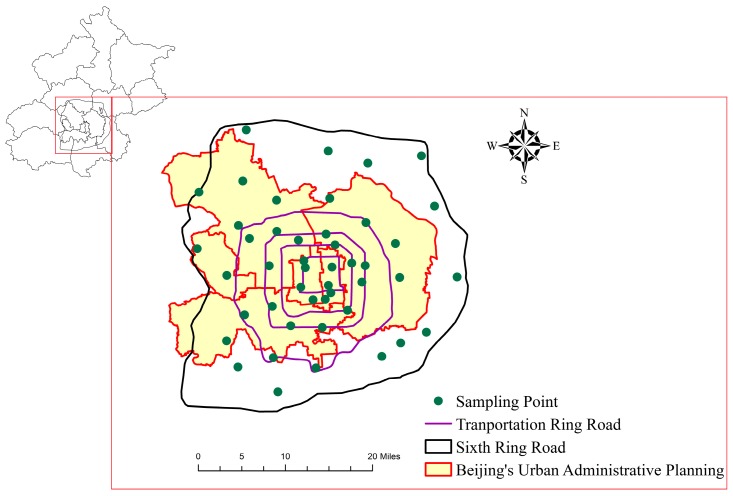
Map showing the distribution of sampling sites.

Forty-six sampling sites were located by GPS within the sixth ring road in Beijing, which were collected on 10 March 2014. Topsoil samples between zero and five centimeters were collected near the relatively stable zones, such as schools, parks, and green belts. At each sampling site, five sub-samples were collected (approximately 16 m^2^) using stainless steel shovel. These sub-samples were thoroughly mixed into a composite sample, with a total sample size of one kilogram. The soil samples were stored in plastic bags and transferred to the laboratory for further analysis. The location of sampling sites is shown in Figure 1.

### 2.2. Laboratory Analysis

After the air-drying and grinding process, the soil samples were acid (HNO_3_-HF-HClO_4_) digested. Then, the concentrations of twelve metals (Nickel, Ni, Lithium, Li, Vanadium, V, Cobalt, Co, Barium, Ba, Strontium, Sr, Chrome, Cr, Molybdenum, Mo, Copper, Cu, Cadmium, Cd, Zinc, Zn, Lead, Pb) were analyzed by Inductively Coupled Plasma Optical Emission Spectrometry (ICP-OES). The procedural blank, duplicate analysis and standard reference materials (GBW) were included for quality assurance and control (QA/QC). The accuracy of standard reference material (GBW) was <8%. The relative standard deviation (RSD) of duplicate samples was below 6%.

### 2.3. Numerical Analysis

#### 2.3.1. Enrichment Factor

Enrichment factor is an important index to describe the influence of human activity on the enrichment of heavy metals in soil and sediment [24]. In order to minimize the error from the process of sampling and handling, the metal concentrations were normalized by the reference element. In general, stable elements—such as Scandium (Sc), Aluminum (Al), and Iron (Fe)—were selected as the reference elements [25]. The calculation is as follows:

(1) $$\text{EF}=\frac{{\left[C_{i}/C_{n}\right]}_{\text{sample}}}{{\left[C_{i}/C_{n}\right]}_{\text{background}}}$$

where ${\left[C_{i}/C_{n}\right]}_{\text{sample}}$ is the ratio of the measured concentration of one metal element in the topsoil with that of reference element. ${\left[C_{i}/C_{n}\right]}_{\text{background}}$ is the ratio of the background concentration of one metal element in the topsoil with that of reference element. This study chose Scandium (Sc) as the reference element. In addition, the background values of twelve metals in Beijing were chosen [26]. The background value of Scandium (Sc) is 10 mg·kg^−1^. According to the variety of enrichment factors, the degree of contamination was divided into six grades, which are listed in Table 1.

#### 2.3.2. Statistical Analysis

Principal component analysis (PCA) is a mathematical procedure that has been widely used in source apportionment of soil, atmosphere, and water [27,28,29]. Topsoil is mainly influenced by soil parent material and human activity during the formation process. The constitution feature of elements can be investigated by Principle Component Analysis (PCA) [30]. This method can also reduce the spatial plurality in order to provide evidence to trace the sources [31]. Cluster analysis (CA) is an efficient method for judging the source of metal elements. Cluster distance refers to the intimating degree between elements and represents their relationship [32]. The results of PCA can be examined effectively by cluster analysis [33]. In this study, PCA and CA were carried out on the concentrations of twelve metals in topsoil using SPSS 19.0 (IBM Co., Armonk, NY, USA).

#### 2.3.3. Human Health Risk Assessment

There are three routes of exposure for soil metals to the human body: (a) Dose contacted through ingestion (${\text{AD}}_{\text{ing}}$); (b) Dose contacted through inhalation (${\text{AD}}_{\text{inh}}$); (c) Dose absorbed through dermal contact (${\text{AD}}_{\text{der}}$). According to the method of health risk assessment proposed by the United States environmental protection agency (EPA) [34,35,36], the computation formulae are as follows:

(2) $${\text{AD}}_{\text{ing}}=\frac{C\times {\text{IR}}_{\text{ing}}\times \text{CF}\times \text{EF}\times \text{ED}}{\text{BW}\times \text{AT}}$$

(3) $${\text{AD}}_{\text{inh}}=\frac{C\times {\text{IR}}_{\text{inh}}\times \text{EF}\times \text{ED}}{\text{PEF}\times \text{BW}\times \text{AT}}$$

(4) $${\text{AD}}_{\text{der}}=\frac{C\times \text{CF}\times \text{SA}\times \text{AF}\times \text{ABS}\times \text{EF}\times \text{ED}}{\text{BW}\times \text{AT}}$$

C (mg·kg^−1^) is the concentration of trace element in topsoil; IR_ing_ (mg·d^−1^) is the ingestion rate; IR_inh_ (m^3^·d^−1^) is inhalation rate; CF (kg·mg^−1^) is the conversion factors; EF (d·a^−1^) is the exposure frequency; ED (a) is the exposure duration; PEF (m^3^·kg^−1^) is the particle emission factor; SA is the exposed skin area (cm^2^); AF (mg·cm^−1^·d^−1^) is the skin adherence factor; ABS (unitless) is the dermal absorption factor; BW (kg) is the average body weight; AT (d) is the averaging time. The reference values for all parameters are listed in Table 2.

Health risk assessment is divided into non-carcinogenic risk assessment and carcinogenic risk assessment:

(1) Non-carcinogenic risk assessment:

(5) $${\text{HQ}}_{i}=\frac{{\text{AD}}_{\text{ij}}}{{\text{RfD}}_{\text{ij}}}$$

(6) $$\text{HI}=\overset{n}{\underset{i=1}{\sum }}{\text{HQ}}_{i}$$

(7) $$\text{HIt}={\text{HI}}_{\text{ing}}+{\text{HI}}_{\text{inh}}+{\text{HI}}_{\text{der}}$$

Hazard quotient (HQ) is the non-carcinogenic risk, where a value of one refers to the threshold reference value suggested by USEPA. RfD (mg·kg^−1^·d^−1^) is the reference dose of metal through oral ingestion, dermal contact, and inhalation of soil particles. HI (Hazard Index) is the total non-carcinogenic hazard quotient of more than one metal element [37]. HIt is the total Hazard Index. HI_ing_, HI_inh_, and HI_der_ refer to the hazard quotients of metals through oral ingestion, inhalation, and dermal contact, respectively. In cases that the non-carcinogenic HQ does not exceed the threshold (HQ below one), no potential non-carcinogenic risks occur in the study area.

(2) Carcinogenic risk assessment:

(8) $$\text{Risk}={\text{AD}}_{\text{ij}}\times {\text{SF}}_{\text{ij}}$$

Risk is of an individual developing cancer over a lifetime (unitless) and SF (mg/kg-day) is the slope factor of a carcinogen. The reference values for all parameters are listed in Table 3. The acceptable risk value is in the range of 10^−6^–10^−4^ [38].

## 3. Results and Discussion

### 3.1. Spatial Distribution of TopSoil Metals

**Figure 2:**
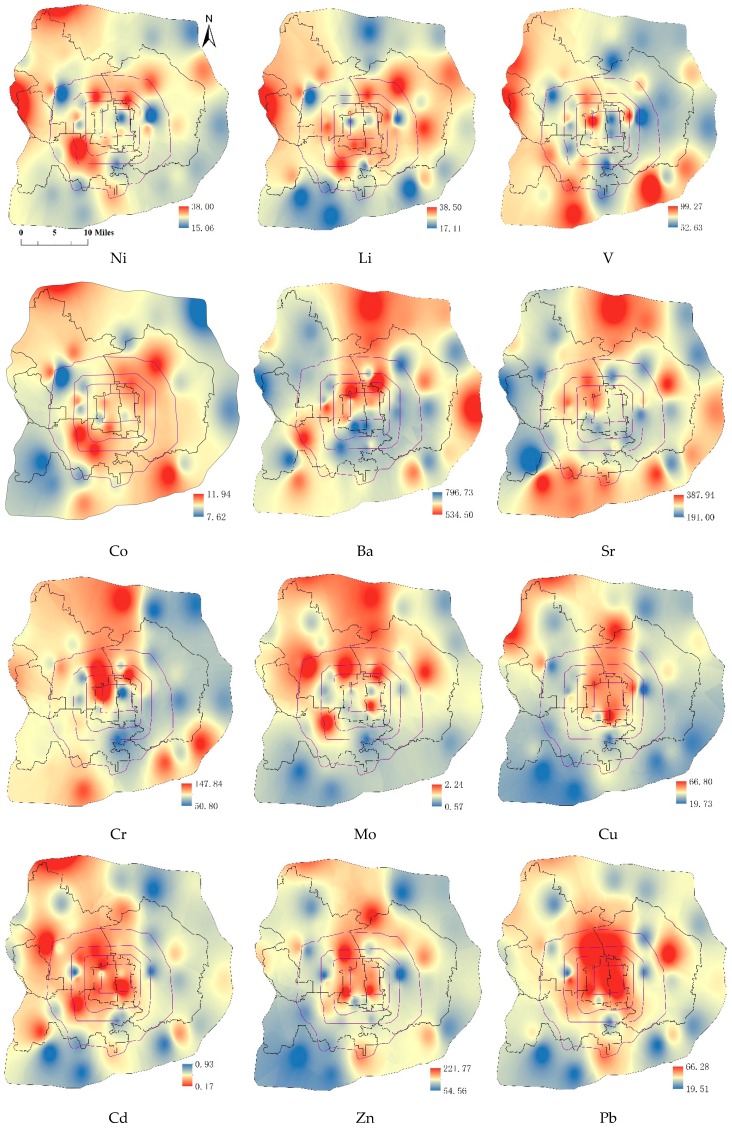
Spatial distribution of metal element concentrations in the topsoil (mg/kg).

Figure 2 shows the spatial distribution patterns of twelve metals (Ni, Li, V, Co, Ba, Sr, Cr, Mo, Cu, Cd, Zn, Pb) inside the sixth ring road of Beijing. In addition, descriptive statistics of metal in the topsoil is presented in Table 4. The distribution of Ni presents that the low concentration area is mainly distributed between the south fifth ring road and the south sixth ring road. The high concentration of Ni appears in the center of the Fengtai District and the area near the northwest edge. Land use types of these areas include forest and urban construction land. The average concentration of Ni is 25.09 mg·kg^−1^, which is a little higher than the background concentrations of Beijing (BCB) and lower than the background concentrations of national (BCN). The hot-spot is located in the junction of the Mentougou District and the Shijingshan District (38.00 mg·kg^−1^). The concentration of Li was low both in the north area and between south fifth ring road and south sixth ring road, while it was high of most area inside the urban zone. The average concentration of Li is 28.07 mg·kg^−1^, which is lower than the BCN and 2.07 mg·kg^−1^ higher than the BCB. The hot-spot is located in the same regions as Ni (38.50 mg·kg^−1^). The spatial distribution of V appears high in the west and low in the east. It appeared to have high concentration both on the west edge of urban zone and between south fifth ring road and south sixth ring road. The average concentration of V is 75.48 mg·kg^−1^, which is higher than the BCB and lower than the BCN. The average concentration of Co is 10.16 mg·kg^−1^, which is close to the BCB. Its low concentration zone is in the northeast and southwest, and the concentration is rather high elsewhere. The spatial distribution patterns of Ba and Sr are quite consistent. They are low in most places within the fifth ring road and high in north and east outside the fifth ring road. Both Ba and Sr are second main group elements and have similar characteristics. The only difference is between the south fifth and sixth ring road. The average concentrations of Ba and Sr are 612.59 mg·kg^−1^ and 291.00 mg·kg^−1^, both of which are higher than the BCB and BCN. The concentration of Cr is high in the western area and low in most of eastern area. Its average concentration is 75.36 mg·kg^−1^, which is higher than BCB and BCN. The spatial distribution of Mo appears high in the north and low in the south. Its average concentration is 1.16 mg·kg^−1^ higher than the BCB and BCN. The spatial distribution of Cu, Cd, and Zn are quite similar. The high concentration zone is located in the center and northwest of Beijing. Their average concentrations are 38.68 mg·kg^−1^, 0.44 mg·kg^−1^, and 126.22 mg·kg^−1^, which are much higher than the BCB and BCN. Pb shows serious pollution characteristics in the center and trends lighter to its surroundings. Its average concentration is 34.40 mg·kg^−1^, higher than the BCB and BCN.

### 3.2. Source Appointment of Heavy Metal Elements

#### 3.2.1. The Enrichment of Heavy Metal Elements

As shown in Table 5, the enrichment degree of metals in the topsoil can be divided into three types. The average enrichment factors of V, Cr, Co, Ni, Li, Sr, and Ba are below two, which is close to Beijing’s soil background value. Most of the sampling points are minimal enrichment or not at all. Among these seven elements, the enrichment degrees of V and Co in all sampling locations appear to be minimal enrichment. Almost 8.75% of Cr sampling spots are moderate enrichment, while others are minimal enrichment. The average enrichment degrees of Cu, Zn, Pb, and Mo are between two and five (moderately enrichment), indicating that they are being seriously polluted by human activity. The average enrichment degree of Cd is 5.94; 65.71% of samplings are ranked as significant enrichment and 34.29% of the sampling spots are moderately enrichment. 

#### 3.2.2. Principal Component Analysis

The results of the PCA for twelve metals are presented in Table 6. This study uses PCA to analyze through Kaiser standard orthogonal iteration. On the premise that the eigenvalue exceeds one after iteration and the accumulated variance contribution rate is greater than 81.88%, four factors are extracted. These factors contribute 32.80%, 20.69%, 17.74%, and 10.65% to the cumulative variance, respectively. The first principal component includes Pb, Zn, Cd, Cu, and Mo for comparatively high positive loadings. As Pb, Zn, and Cu are usually called traffic pollution recognition elements, factor one is called “traffic index”. What’s more, Co, V, Ni, and Li have positive loads in factor two. From Table 1, we can see that the average concentrations of those four elements are close to the background values. So, it can be inferred that these elements come from soil parent materials. Based on this, factor two is called the “natural index”. Moreover, factor three consists of Sr and Ba, which are in the same main group with similar characteristics. Ba also has a high rate in index one, meaning Ba in the soil is influenced by both nature and human activity. There is only Cr in factor four, which has a high load (0.86). Cr is mainly accumulated in the topsoil and hardly moves to deeper layers. Industrial pollutants and waste gas from coal burning are the main reasons for the high concentration of Cr. Based on the spatial distribution pattern and the sampling period (coal-burning period), we can infer that the contamination of Cr is related to coal burning. Thus, factor four is called the “coal burning index”. The high loads, both in factor one and four of Mo indicate that it is influenced by-various factors.

#### 3.2.3. Clustering Analysis

**Figure 3:**
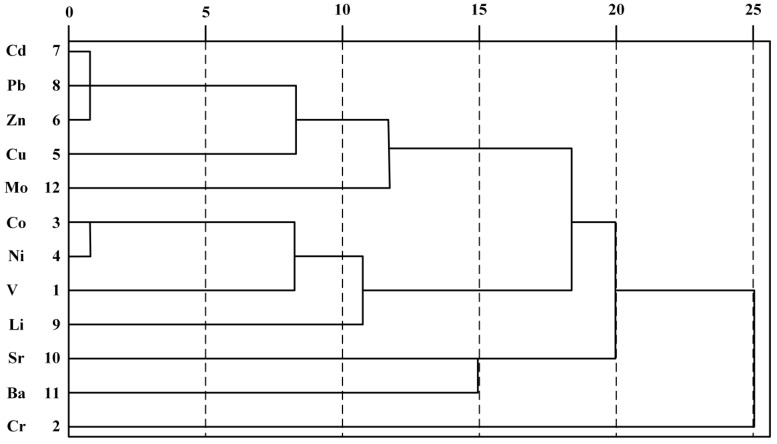
Hierarchical cluster analysis of metals in the topsoil.

The results of CA are shown in Figure 3. This study divides all elements into four types at the class distance of 18. The first class includes Cu, Mo, Pb, Zn, and Cd, and the latter three elements as sub-classes, which are mainly related to the transportation process. The second class includes Co, V, Ni, and Li. Among which Co, V, and Ni are in the same period of the periodic table of the elements. Co and Ni showed identical characteristics as they both belong to the iron series elements and black metals, which means a closer relationship. Class three consists of Sr and Ba. Cr is the only element in class four. Thus, the result of cluster analysis is in accordance with principal component analysis. 

### 3.3. Human Health Risk Assessment

The human health risk assessment of nine heavy metals (Nickel, Ni, Vanadium, V, Cobalt, Co, Cr, Molybdenum, Mo, Copper, Cu, Cadmium, Cd, Zinc, Zn, Lead, Pb) (Table 7) shows that the exposure risk of nine elements through ingestion, inhalation, and dermal contact pathways are below one, indicating that their non-carcinogenic risks are all under the safety standard. What’s more, the non-carcinogenic risks of nine elements vary through different exposure pathways. The pathways of Cr, Ni, Cu, Zn, Pb, and Mo have the sequence that ingestion is first, dermal contact is second, and inhalation is third. V and Cd decreased in the following order: dermal contact > ingestion > inhalation. In addition, the non-carcinogenic risk of Co by ingestion is the highest, followed by inhalation and dermal contact. The non-carcinogenic risks of Cr by three pathways rank first among all metals. The excessive intake of Cr may lead to diarrhea, skin eczema, rhinitis, and bronchitis. Thus, Cr pollution in topsoil should be given serious attention. The HI through ingestion pathways is the highest, and inhalation is lowest. The HIt of each element is ranked as Cr > V > Pb > Ni > Cu > Cd > Co > Zn > Mo. We assessed the carcinogenic risks of Cd, Co, Cr, and Ni for their high carcinogenic effects on human beings. From Table 7, we can see that their risks decreased in the following order: Cr > Co > Ni > Cd. Among them, the high risk index of Cr is 2.5 × 10^−8^, which is within the safety level.

## 4. Conclusions

This study carried out the spatial distribution of twelve metals (Ni, Li, V, Co, Ba, Sr, Cr, Mo, Cu, Cd, Zn, and Pb) using geostatistical methods. It showed that there is a significant spatial variation. The concentrations of Ni, Li, V and Co within the sixth ring road are close to background concentrations of Beijing. Except for few sampling spots, their concentrations are all distributed uniformly and had little spatial variation. Ba and Sr are main group elements and have similar characteristics and spatial distribution patterns. The concentration of Cr was high in the west and low in the east, while that of Mo was high in the north and low in the south. Among twelve elements, the concentrations of Cu, Cd, Zn, and Pb are high in most places of the urban district. Source analysis showed that the accumulation of Ni, Li, V, and Co are mainly influenced by parent material, while Cr, Mo, Cu, Cd, Zn and Pb are mainly affected by human activities, including coal heating, transportation, and industry. Ba and Sr are influenced by both natural and artificial impacts. Nine heavy metals were absorbed by human beings through ingestion, inhalation, and dermal contact pathways. Each exposure pathway has different non-carcinogenic risk. The highest non-carcinogenic risk is the ingestion pathway, which is 1.8 times and 101 times the non-carcinogenic risk of dermal contact and inhalation, respectively. The carcinogenic risks of Cd, Co, Cr, and Ni lay within the safety standard.

## Figures and Tables

**Table 1:** Classification of enrichment factors (EF).

Variation Range	Degree of Contamination
EF ≤ 1	No enrichment
1 < EF ≤ 2	Minimal enrichment
2 < EF ≤ 5	Moderate enrichment
5 < EF ≤ 20	Significant enrichment
20 < EF ≤ 40	Very high enrichment
EF > 40	Extraordinary high enrichment

**Table 2:** Parameter values in average daily dose calculation models (adult).

Parameter	IR_ing_ (mg·d^−1^)	IR_inh_ (m^3^·d^−1^)	CF(mg·kg^−1^)	EF (d·a^−1^)	ED (a)	SA (cm^2^)	AF (mg·cm^−1^·d^−1^)	ABS	PEF (m^3^·kg^−1^)	BW (kg)	AT (d)
value	100	15	1 × 10^−6^	87.5	6	5700	0.2	0.001	1.36 × 10^9^	55.9	ED × 365 (non-carcinogen) 70 × 365 (carcinogen)

IR_ing_: ingestion rate; IR_inh_: inhalation rate; CF: conversion factors; EF: exposure frequency; ED: exposure duration; PEF: particle emission factor; SA: exposed skin area; AF: skin adherence factor; ABS (unitless): dermal absorption factor; BW: average body weight; AT: averaging time.

**Table 3:** References dose (RfD) for non-carcinogenic metals and slope factors for carcinogenic metals.

Element	V	Cr	Co	Ni	Cu	Zn	Cd	Pb	Mo
RfD_ing_	7 × 10^−3^	3 × 10^−3^	2 × 10^−2^	2 × 10^−2^	4 × 10^−2^	3 × 10^−1^	1 × 10^−3^	3.5 × 10^−3^	5 × 10^−3^
RfD_inh_	7 × 10^−3^	2.86 × 10^−5^	5.71 × 10^−6^	2.06 × 10^−2^	4.02 × 10^−2^	3 × 10^−1^	1 × 10^−3^	3.52 × 10^−3^	4.95 × 10^−3^
RfD_der_	7 × 10^−5^	6 × 10^−5^	1.6 × 10^−2^	5.40 × 10^−3^	1.2 × 10^−2^	6 × 10^−2^	1 × 10^−5^	5.25 × 10^−4^	1.9 × 10^−3^
SF_inh_	-	42	9.8	0.84	-		6.3	-	-

RfD_ing_: reference dose for ingestion; RfD_inh_: reference dose for inhalation; RfD_der_: reference dose for dermal contact; SF_inh_: slope factor for inhalation route.

**Table 4:** Descriptive statistics of metal in the topsoil of the study area (mg·kg^−1^).

Element	V	Cr	Co	Ni	Cu	Zn	Cd	Pb	Li	Sr	Ba	Mo
Max	99.27	147.84	11.94	38	66.8	221.77	0.93	66.28	38.5	387.94	796.73	2.24
Min	52.63	50.8	7.62	15.06	19.73	54.2	0.17	19.51	17.11	191	534.5	0.57
Mean	75.48	75.36	10.16	25.09	38.68	126.22	0.44	34.4	28.07	291	612.59	1.16
Beijing’s Background	71	58	10	25	20	58	0.09	19	26	271	598	0.6
National Background	87	73	13	29	24	68	0.11	23	37	156	495	0.7

**Table 5:** Proportion of contamination classification of metal elements in the topsoil.

Element	Average Enrichment Coefficient	Contamination Classification Proportion			
		EF ≤ 1	1 < EF ≤ 2	2 < EF ≤ 5	5 < EF ≤ 20
V	1.29	0.00%	100.00%	0.00%	0.00%
Cr	1.58	0.00%	91.43%	8.57%	0.00%
Co	1.23	0.00%	100.00%	0.00%	0.00%
Ni	1.22	11.43%	88.57%	0.00%	0.00%
Cu	2.35	0.00%	38.64%	61.36%	0.00%
Zn	2.64	0.00%	25.71%	68.57%	5.71%
Cd	5.94	0.00%	0.00%	34.29%	65.71%
Pb	2.2	0.00%	32.35%	64.71%	2.94%
Li	1.31	5.71%	94.29%	0.00%	0.00%
Sr	1.3	5.71%	94.29%	0.00%	0.00%
Ba	1.24	5.88%	94.12%	0.00%	0.00%
Mo	2.35	0.00%	48.57%	48.57%	2.86%

**Table 6:** Rotated component matrix of metals in the topsoil.

Element	Factor			
	1	2	3	4
Pb	**0.93**	−0.01	−0.06	0.00
Zn	**0.91**	0.13	0.07	0.07
Cd	**0.91**	0.05	−0.16	0.17
Cu	**0.78**	0.08	0.17	−0.13
Mo	**0.58**	−0.03	0.08	0.56
Co	0.14	**0.95**	−0.17	0.08
V	−0.20	**0.84**	0.16	−0.17
Ni	0.34	**0.76**	−0.36	0.25
Li	0.22	**0.51**	−0.68	−0.23
Sr	−0.06	0.01	**0.89**	0.10
Ba	0.48	−0.09	**0.78**	−0.07
Cr	−0.05	0.02	0.09	**0.86**
Eigenvalue	3.94	2.48	2.13	1.28
Contribution rate (%)	32.80	20.69	17.74	10.65
Accumulating contribution rate (%)	32.80	53.48	71.22	81.88

**Table 7:** Hazard quotient (HQ) and carcinogenic risk for each metal and pathway in the urban topsoil.

		V	Cr	Co	Ni	Cu	Zn	Cd	Pb	Mo	HI
HQ_ing_	Max	6.08 × 10^−3^	2.11 × 10^−2^	2.56 × 10^−4^	8.15 × 10^−4^	7.16 × 10^−4^	3.17 × 10^−4^	3.99 × 10^−4^	8.12 × 10^−3^	1.92 × 10^−4^	3.80 × 10^−2^
	Min	3.22 × 10^−3^	7.26 × 10^−3^	1.63 × 10^−4^	3.23 × 10^−4^	2.12 × 10^−4^	7.75 × 10^−^^5^	7.29 × 10^−^^5^	2.93 × 10^−3^	4.89 × 10^−5^	1.38 × 10^−2^
	Mean	4.62 × 10^−3^	1.08 × 10^−2^	2.18 × 10^−4^	5.38 × 10^−4^	4.15 × 10^−4^	1.80 × 10^−4^	1.88 × 10^−4^	4.21 × 10^−3^	9.92 × 10^−5^	2.12 × 10^−2^
HQ_inh_	Max	6.71 × 10^−^^7^	2.45 × 10^−^^4^	9.89 × 10^−^^5^	8.73 × 10^−^^8^	7.86 × 10^−^^8^	3.50 × 10^−^^8^	4.40 × 10^−^^8^	8.91 × 10^−^^7^	2.14 × 10^−^^8^	3.45 × 10^−^^4^
	Min	3.56 × 10^−^^7^	8.40 × 10^−^^5^	6.31 × 10^−^^5^	3.46 × 10^−^^8^	2.32 × 10^−^^8^	8.55 × 10^−^^9^	8.04 × 10^−^^9^	2.62 × 10^−^^7^	5.45 × 10^−^^9^	1.48 × 10^−^^4^
	Mean	5.10 × 10^−^^7^	1.25 × 10^−^^4^	8.41 × 10^−^^5^	5.76 × 10^−^^8^	4.55 × 10^−^^8^	1.99 × 10^−^^8^	2.07 × 10^−^^8^	4.62 × 10^−^^7^	1.11 × 10^−^^8^	2.10 × 10^−^^4^
HQ_der_	Max	6.93 × 10^−3^	1.20 × 10^−2^	3.65 × 10^−6^	3.44 × 10^−5^	2.72 × 10^−5^	1.81 × 10^−5^	4.55 × 10^−4^	6.17 × 10^−4^	5.76 × 10^−6^	2.01 × 10^−2^
	Min	3.68 × 10^−3^	4.14 × 10^−3^	2.33 × 10^−6^	1.36 × 10^−5^	8.04 × 10^−6^	4.42 × 10^−6^	8.31 × 10^−5^	1.82 × 10^−4^	1.47 × 10^−6^	8.11 × 10^−3^
	Mean	5.27 × 10^−3^	6.14 × 10^−3^	3.10 × 10^−6^	2.27 × 10^−5^	1.58 × 10^−5^	1.03 × 10^−5^	2.14 × 10^−4^	3.20 × 10^−4^	2.98 × 10^−6^	1.20 × 10^−2^
HIt	Max	1.30 × 10^−2^	3.34 × 10^−2^	3.59 × 10^−4^	8.49 × 10^−4^	7.43 × 10^−4^	3.35× 10^−4^	8.54 × 10^−4^	8.74 × 10^−3^	1.98 × 10^−4^	5.85 × 10^−2^
	Min	6.9 × 10^−3^	1.15 × 10^−2^	2.29 × 10^−4^	3.37 × 10^−4^	2.20 × 10^−4^	8.19 × 10^−5^	1.56 × 10^−4^	2.57 × 10^−3^	5.04 × 10^−5^	2.20 × 10^−2^
	Mean	9.9 × 10^−3^	1.70 × 10^−2^	3.05 × 10^−4^	5.61 × 10^−4^	4.30 × 10^−4^	1.91 × 10^−4^	4.03 × 10^−4^	4.54 × 10^−3^	1.02 × 10^−4^	3.34 × 10^−2^
risk	Max		2.5 × 10^−8^	4.7 × 10^−10^	1.3 × 10^−10^			2.4 × 10^−11^			
	Min		8.7 × 10^−9^	3 × 10^−10^	5.1 × 10^−11^			4.3 × 10^−12^			
	Mean		1.3 × 10^−8^	4 × 10^−10^	8.5 × 10^−11^			1.1 × 10^−11^			

HQ_ing_: hazard quotient, ingestion; HQ_inh_: hazard quotient, inhalation; HQ_der_: hazard quotient, dermal contact; HIt: total hazard index.

## References

[1] Zhao L., Xu Y., Hou H., Shangguan Y., Li F. (2014). Source identification and health risk assessment of metals in urban soils around the Tanggu chemical industrial district, Tianjin, China. Sci. Total Environ..

[2] Pan L.B., Ma J., Wang X.L., Hou H. (2016). Heavy metals in soils from a typical county in Shanxi Province, China: Levels, sources and spatial distribution. Chemosphere.

[3] Qing X., Yutong Z., Shenggao L. (2015). Assessment of heavy metal pollution and human health risk in urban soils of steel industrial city (Anshan), Liaoning, Northeast China. Int. J. Ecotoxicol. Environ. Saf..

[4] Zhang C., Yang Y., Li W., Zhang C., Zhang R., Mei Y., Liao X., Liu Y. (2015). Spatial distribution and ecological risk assessment of trace metals in urban soils in Wuhan, central China. Environ. Monit. Assess..

[5] Rodríguez-Seijo A., Andrade M.L., Vega F.A. (2015). Origin and spatial distribution of metals in urban soils. J. Soils Sediments.

[6] Zhou M., Lv Y., Shen R., Zhou Z., Zhou J., Hu S., Zhou X. (2015). Assessment of heavy metal pollution in surface soils of Hankou region in Wuhan, China. Geo-Informatics in Resource Management and Sustainable Ecosystem.

[7] Xu L., Lu A., Wang J., Ma Z., Pan L., Feng X. (2016). Effect of land use type on metals accumulation and risk assessment in soil in the peri-urban area of Beijing, China. Hum. Ecol. Risk Assess. Int. J..

[8] Shen R., Li J., Yang M., Zeng M., Zhou M. (2015). Spatial distribution of heavy metals in roadside soils based on voronoi diagram: A case study of Wuhan city. Geo-Informatics in Resource Management and Sustainable Ecosystem.

[9] Ordóñez A., Álvarez R., De Miguel E., Charlesworth S. (2015). Spatial and temporal variations of trace element distribution in soils and street dust of an industrial town in NW Spain: 15 years of study. Sci. Total Environ..

[10] Quan S.X., Yan B., Yang F., Li N., Xiao X.M., Fu J.M. (2015). Spatial distribution of heavy metal contamination in soils near a primitive e-waste recycling site. Environ. Sci. Pollut. Res. Int..

[11] Jin Z., Li Z., Li Q., Hu Q., Yang R., Tang H., Li M., Huang B., Zhang J., Li G. (2015). Canonical correspondence analysis of soil heavy metal pollution, microflora and enzyme activities in the Pb–Zn mine tailing dam collapse area of Sidi village, SW China. Environ. Earth Sci..

[12] Elbana T.A., Ramadan M.A., Gaber H.M., Bahnassy M.H., Kishk F.M., Selim H.M. (2013). Heavy metals accumulation and spatial distribution in long term wastewater irrigated soils. J. Environ. Chem. Eng..

[13] Chen H., Teng Y., Lu S., Wang Y., Wu J., Wang J. (2016). Source apportionment and health risk assessment of trace metals in surface soils of Beijing metropolitan, China. Chemosphere.

[14] Li X., Liu L., Wang Y., Luo G., Chen X., Yang X., Myrna H.P.H., Guo R., Wang H., Cui J. (2013). Heavy metal contamination of urban soil in an old industrial city (Shenyang) in Northeast China. Geoderma.

[15] Qu M.K., Li W.D., Zhang C.R., Wang S.Q., Yang Y., He L.Y. (2013). Source apportionment of heavy metals in soils using multivariate statistics and geostatistics. Pedosphere.

[16] Ali Z., Malik R.N., Shinwari Z.K., Qadir A. (2015). Enrichment, risk assessment, and statistical apportionment of heavy metals in tannery-affected areas. Int. J. Environ. Sci. Technol..

[17] Dartan G., Taşpınar F., Toröz İ. (2015). Assessment of heavy metals in agricultural soils and their source apportionment: A Turkish district survey. Environ. Monit. Assess..

[18] Karim Z., Qureshi B.A. (2015). Health risk assessment of heavy metals in urban soil of Karachi, Pakistan. Hum. Ecol. Risk Assess. Int. J..

[19] Wei J., Chen M., Song J., Luo F., Han L., Li C., Dong M. (2015). Assessment of human health risk for an area impacted by a large-scale metallurgical refinery complex in Hunan, China. Hum. Ecol. Risk Assess. Int. J..

[20] De Miguel E., Iribarren I., Chacon E., Ordonez A., Charlesworth S. (2007). Risk-based evaluation of the exposure of children to trace elements in playgrounds in Madrid (Spain). Chemosphere.

[21] Fan S. (2015). Pollution and health risk assessment of heavy metals in soil around a smelter in Changqing town of Baoji City. Environ. Eng..

[22] Guo P.R., Lei Y.Q., Zhou Q.L., Wang C., Pan J.C. (2015). Distribution characteristics of heavy metals in environmental samples around electroplating factories and the health risk assessment. Huan Jing Ke Xue.

[23] Zhao P., Lu X.W., Huang L., Zhuang S.K., Shi C.Q., Yin N., Wang Q., Li Y.X., Zhu Y.J. (2015). Pollution level and health risk of heavy metals in dust from city parks of Xi’an. Int. J. Urban Environ. Urban Ecol..

[24] Zoller W.H., Gladney E.S., Duce R.A. (1974). Atmospheric concentrations and sources of trace metals at the South Pole. Science.

[25] Zhang X.Z., Bao Z.Y., Tang J.H. (2006). Application of the enrichment factor in evaluating of heavy metals contamination in the environmental geochemistry. Geo Sci. Technol. Inf..

[26] Cheng H.X., Li K., Li M., Yang K., Liu F., Cheng X. (2014). Geochemical background and baseline value of chemical elements in urban soil in China. Earth Sci. Front..

[27] Yu L., Cheng J., Zhan J., Jiang A. (2016). Environmental quality and sources of heavy metals in the topsoil based on multivariate statistical analyses: A case study in Laiwu City, Shandong Province, China. Nat. Hazards.

[28] Gao J., Peng X., Chen G., Xu J., Shi G.L., Zhang Y.C., Feng Y.C. (2016). Insights into the chemical characterization and sources of PM_2.5_ in Beijing at a 1-h time resolution. Sci. Total Environ..

[29] Sukumaran M., Devarayan K. (2016). Evaluation of water quality of Kaveri River in Tiruchirappalli district, Tamil Nadu by principal component analysis. Curr. World Environ..

[30] Facchinelli A., Sacchi E., Mallen L. (2001). Multivariate statistical and GIS-based approach to identify heavy metal sources in soils. Environ. Pollut..

[31] Wiseman C.L., Zereini F., Püttmann W. (2015). Metal and metalloid accumulation in cultivated urban soils: A medium-term study of trends in Toronto, Canada. Sci. Total Environ..

[32] Zhang C. (2006). Using multivariate analyses and GIS to identify pollutants and their spatial patterns in urban soils in Galway, Ireland. Environ. Pollut..

[33] Lee C.S., Li X., Shi W., Cheung S.C., Iain T. (2006). Metal contamination in urban, suburban, and country park soils of Hong Kong: A study based on GIS and multivariate statistics. Sci. Total Environ..

[34] USEPA (U.S. Environmental Protection Agency) (1989). Risk Assessment Guidance for Superfund: Human Health Evaluation Manual.

[35] USEPA (U.S. Environmental Protection Agency) (2001). Risk Assessment Guidance for Superfund: Process for Conducting Probabilistic Risk Assessment.

[36] USEPA (U.S. Environmental Protection Agency) (2004). Risk Assessment Guidance for Superfund: Human Health Evaluation Manual.

[37] Ferreira-Baptista L., De Miguel E. (2005). Geochemistry and risk assessment of street dust in Luanda, Angola: A tropical urban environment. Atmos. Environ..

[38] Liu Y., Lei S., Chen X. (2016). Assessment of heavy metal pollution and human health risk in urban soils of the coal mining city, Huainan, East China. Hum. Ecol. Risk Assess..

